# Simple Methods of Determining Confidence Intervals for Functions of Estimates in Published Results

**DOI:** 10.1371/journal.pone.0098498

**Published:** 2014-05-28

**Authors:** Garrett Fitzmaurice, Stuart Lipsitz, Sundar Natarajan, Atul Gawande, Debajyoti Sinha, Caprice Greenberg, Edward Giovannucci

**Affiliations:** 1 McLean Hospital Laboratory for Psychiatric Biostatistics, Belmont, Massachusetts, United States of America; 2 Division of Internal Medicine, Brigham and Women's Hospital, Boston, Massachusetts, United States of America; 3 Ariadne Labs, Boston, Massachusetts, United States of America; 4 Department of Medicine, New York University School of Medicine, New York, New York, United States of America; 5 Department of Statistics, Florida State University, Tallahassee, Florida, United States of America; 6 Department of Surgery, University of Wisconsin, Madison, Wisconsin, United States of America; 7 Departments of Nutrition and Epidemiology, Harvard School of Public Health, Boston, Massachusetts, United States of America; University of Pennsylvania, United States of America

## Abstract

Often, the reader of a published paper is interested in a comparison of parameters that has not been presented. It is not possible to make inferences beyond point estimation since the standard error for the contrast of the estimated parameters depends upon the (unreported) correlation. This study explores approaches to obtain valid confidence intervals when the correlation 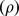 is unknown. We illustrate three proposed approaches using data from the National Health Interview Survey. The three approaches include the Bonferroni method and the standard confidence interval assuming 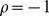 (most conservative) or 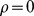 (when the correlation is known to be non-negative). The Bonferroni approach is found to be the most conservative. For the difference in two estimated parameter, the standard confidence interval assuming 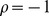 yields a 95% confidence interval that is approximately 12.5% narrower than the Bonferroni confidence interval; when the correlation is known to be positive, the standard 95% confidence interval assuming 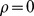 is approximately 38% narrower than the Bonferroni. In summary, this article demonstrates simple methods to determine confidence intervals for unreported comparisons. We suggest use of the standard confidence interval assuming 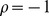 if no information is available or 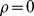 if the correlation is known to be non-negative.

## Introduction

The conventional presentation of measures of association or effect in medical journals is in terms of tables of estimates and standard errors. Unfortunately, this information alone does not allow readers to make inference on a comparison of interest that has not been presented. Although point estimation of the contrast is straightforward, inference is not because its standard error depends upon unreported correlations among the published estimates. There can be substantial correlation among the estimates due to the study design (e.g., clustering in complex sample surveys), the method of estimation (e.g., adjusted estimates that control for confounding), or comparisons with a common reference group.

For example, a recent article on PSA screening, using data from the 2000 and 2005 National Health Interview Survey (NHIS), presents unadjusted estimates and confidence intervals, of the US population screening rates for men ≥70 years old in three distinct life expectancy groups [1]. However, these results do not permit inferences about comparisons among the three groups. The unadjusted estimates of the population PSA screening rates in the three groups are correlated due to the complex sampling frame utilized in these surveys with stratification and clustering. Because there are individuals from the same cluster in all three groups, and observations from the same cluster tend to be positively correlated, the unadjusted estimates of PSA screening rates in the three groups are positively correlated. To make inference on the differences in the screening rates in the three groups, we require standard errors for the differences. However, these depend not only on the standard errors for the estimated rates but also on the unreported correlations among the estimated rates.

Another common example where conventional presentation of estimates and standard errors does not allow readers to make inference on a comparison of interest is the reporting of effects of categorical covariates in regression models. For example, in the PSA screening study, the authors also present the results of a logistic regression model for screening rates, where one of the key covariates is the life expectancy variable discussed earlier, categorized as 'high', 'intermediate', or 'low', with 'high' being the reference group. Adjusted odds ratios and 95% confidence intervals are presented. Suppose, however, the reader is interested in the odds ratio for 'intermediate' versus 'low'. An estimate can be obtained by taking the ratio of the reported odds ratios of 'intermediate' versus 'high' and 'low' versus 'high'; note, the two odds ratios forming this ratio are correlated because they involve a comparison with a common reference group ('high'). However, a confidence interval for this ratio cannot be obtained without information about the (unreported) correlation between the two odds ratios.

This note provides simple, theoretically valid methods to obtain confidence intervals for these measures of effect that will have the correct coverage probabilities, i.e., in repeated sampling from the same population, the proportion of 95% confidence intervals that contain the true value will be at least 95%. For many studies, the typical contrast of most interest is a simple difference in parameters; for example, a difference in rates (or log rates), or a difference in log odds. We describe methods to obtain confidence intervals using differences in correlated estimates. In Section 1 of the Methods we describe the use of the Bonferroni inequality to obtain confidence intervals. In Section 2 of the Methods we discuss the potential conservativeness of the Bonferroni method and consider alternative methods for obtaining less conservative confidence intervals. In the Results, these methods are applied to the PSA screening study.

## Methods

### 1. Bonferroni Method for Difference in Parameters

Most readers of medical journals are familiar with the Bonferroni inequality as applied to multiple tests [2]. In that setting, to preserve an overall 5% chance of finding significant results if K tests are performed, each test is performed at a significance level of 5% divided by K. In this section, we consider differences of two parameters *β*_1_ and *β*_2_ (here K = 2); say Δ =  *β*_1_−*β*_2_. We obtain a confidence interval for Δ by first obtaining separate confidence intervals for *β*_1_ and *β*_2_, and then combining the endpoints of the two confidence intervals. However, by the Bonferroni inequality [3], for the resulting confidence interval for Δ to have 95% coverage probability, we must calculate 97.5% CI's for *β*_1_ and *β*_2_ before combining them.

Specifically, if 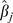, *j* = 1,2, is approximately normally distributed, then a 97.5% CI for *β_j_* is 
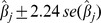
 where 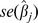 is the estimated standard error of 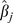 We denote the 97.5% CIs for *β*_1_ and *β*_2_, by 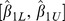 and 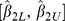, respectively. The lower and upper limits of the Bonferroni 95% CI for the difference in parameters Δ, (Δ_L_, Δ_U_), is 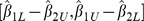
or equivalently 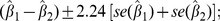 see [4], for example, for a detailed proof of this result.

### 2. Less Conservative Confidence Intervals

The Bonferroni confidence interval has the desirable property that it can be easily calculated, and does not require any knowledge or assumptions about the correlation between 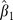 and 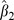. However, Bonferroni confidence intervals are known to be conservative (unnecessarily wide) when many confidence intervals are simultaneously calculated [5]. Although the 95% Bonferroni confidence interval for Δ =  *β*_1_−*β*_2_ is based on only two confidence intervals, it can still be conservative, as we now discuss. Further, we also describe a simple alternative that is less conservative.

Recall that the 95% Bonferroni confidence interval for Δ =  *β*_1_−*β*_2_ is: 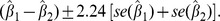



In contrast, a general expression for the standard 95% confidence interval for Δ =  *β*_1_−*β*_2_ is: 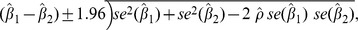
where 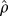 is the (unreported) estimated correlation between 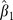 and 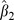. From this expression, note that the standard error for 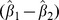 takes on its maximum value, and thus the confidence interval is widest, when 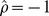, yielding 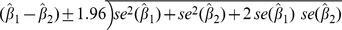.

However, it can easily be shown that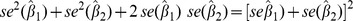,so that a less conservative confidence interval than the Bonferroni interval has the simple form
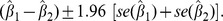



This yields a confidence interval that is 12.5% narrower than the corresponding Bonferroni confidence interval presented earlier, while ensuring coverage probability of at least 95%. We also note that this 95% confidence interval is even more straightforward to calculate because it involves only differences between the reported lower and upper limits of the 95% confidence intervals for *β*_1_ and *β*_2_. That is, if we now denote the 95% CIs for *β*_1_ and *β*_2_ by 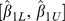 and 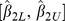, respectively, then the 95% CI for Δ =  *β*_1_−*β*_2_ is simply 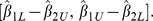


Finally, in the two examples that have motivated this paper, although the value of the correlation between 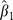 and 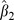 may not be known, it can safely be assumed to be positive, i.e., 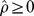. In both of these settings, even less conservative confidence intervals can be obtained by assuming 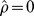. This yields the following 95% confidence interval for Δ =  *β*_1_−*β*_2_: 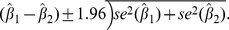



When 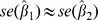 this yields a 95% confidence interval that is approximately 38% narrower than the corresponding Bonferroni confidence interval and 29% narrower than the corresponding confidence interval assuming 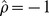. When 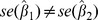 this yields confidence intervals that are anywhere from 12.5% to 38% narrower, i.e., the improvements relative to the Bonferroni method are greater when 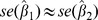. Finally, when the correlation is known (and positive) rather than assumed to be zero, the standard method yields confidence intervals that are 13%, 29%, and 50% narrower than our proposed method for correlations of 0.25, 0.5, and 0.75 respectively when 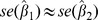; the differences between the methods are more modest when 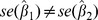. This emphasizes the point that while the proposed method is an improvement over the Bonferroni method, it can be quite conservative when the correlation is appreciable.

## Results

### Application to Prostate-Specific Antigen Screening Study

We apply the proposed method to the results from the PSA screening study [1]. The authors present unadjusted estimates of the US population screening rates for men ≥70 years old in three life expectancy groups: 1) those having high life expectancies (15% probability of 5-year mortality), 2) intermediate life expectancies (16% to 47% probability of 5-year mortality), and 3) low life expectancies (≥48% probability of 5-year mortality). Suppose we are interested in the screening rate differences among groups. The reported estimated rates (95% CIs) are 47.3% (44.0%, 50.6%) for the high life expectancy group; 39.2% (35.9%, 42.4%) for the intermediate life expectancy group; and 30.7% (25.8%, 35.6%) for the low life expectancy group. It is easily seen that these confidence intervals are symmetric about the rates, and thus equal the estimates ±1.96 standard errors. Therefore, the estimated standard errors are 1.7 for the high life expectancy group; 1.7 for the intermediate life expectancy group; and 2.5 for the low life expectancy group, respectively. Recognizing that due to the complex survey design with clustering, the correlation between the rates can safely be assumed to be positive, a 95% confidence interval for the rate difference, say Δ =  *β*_1_−*β*_2_, can be calculated as 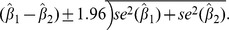 Thus, the screening rate difference of high versus low life expectancy groups is 16.6% (10.7%, 22.5%) and intermediate versus low life expectancy groups is 8.5% (3.8%, 13.2%). In contrast, the more conservative 95% Bonferroni confidence interval for the rate difference, 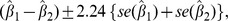 yields discernibly wider confidence intervals: (7.2%, 26.0%) for high versus low life expectancy groups and (−0.9%, 17.9%) for intermediate versus low life expectancy groups.

Further, in the PSA screening study, the authors also present the results of a logistic regression model for screening rates, where one of the key covariates is the life expectancy variable discussed above, categorized as high, intermediate, or low, with 'high' being the reference group. Adjusted odds ratios and 95% confidence intervals are presented. From the paper, the estimated adjusted odds ratio for screening (95% CIs) are 0.81 (0.65, 1.01) for intermediate versus high; and 0.66 (0.48, 0.91) for low versus high. Further, it is easily seen that these confidence intervals were initially obtained on the log odds ratio scale as log OR±1.96 se(log OR), and the endpoints for this CI were exponentiated. Thus, the estimated adjusted log-odds ratio for screening (se) is −0.21 (0.11) for intermediate versus high; and −0.42 (0.16) for low versus high. Suppose we are interested in the odds ratio for intermediate versus low group. Recognizing that the reported estimated adjusted log-odds ratios are positively correlated due to the common reference group for both estimates, the log-odds ratio for intermediate versus low group is −0.21−(−0.42)  = 0.21, with 95% CI (−0.17, 0.59); this 95% CI is based on the expression, 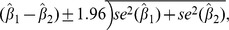 from Section 2 of the Methods (assuming 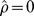). Exponentiating the log odds ratio and the endpoints of the 95% CI, we obtain an adjusted odds ratio (95% CI) for the intermediate versus low group of 1.23 (0.84, 1.81). Note that if one uses the most conservative assumption about the correlation (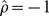) with 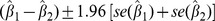 then the 95% CI for the intermediate versus low group, (0.73, 2.09) is wider than under the assumption that 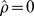, although somewhat narrower than the corresponding 95% Bonferroni confidence interval with 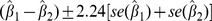, which equals (0.66,2.28).

## Discussion

This article demonstrates simple and theoretically valid methods to determine confidence intervals for comparisons of interest that have not been reported. The main focus is on univariate functions of two parameters, such as the rate difference or relative risk or a regression parameter for a different reference group than published. All of the methods described in this paper are very simple to apply; with the appropriate results abstracted from a published paper, a calculator can be used to obtain the confidence interval and make inferences on a comparison of interest. The methods can also be applied when standard errors based on the bootstrap or other resampling methods have been reported as an alternative to the usual asymptotic standard errors; however, the validity of the methods does require the assumption that the sampling distribution of the estimates is approximately normal. Although the 95% Bonferroni confidence interval is statistically valid, it is conservative. We propose an alternative to the Bonferroni confidence interval using the most conservative assumption about the correlation (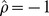), which leads to a less conservative confidence interval. Finally, there are many settings where the value of the correlation between 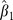 and 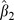 may not be known, but it can safely be assumed to be positive. In those settings, an even less conservative confidence interval can be obtained by assuming zero correlation. Although the proposed method is an improvement over the Bonferroni method, it can be quite conservative when the correlation is appreciable and should only be used when the information required to construct more appropriate confidence intervals is not available.

There is a connection between the proposed method and the approach of testing whether two parameters are different by looking at whether there is overlap between the confidence intervals for the estimates of the parameters [6]. The focus of the latter method is on hypothesis testing, rather than the construction of confidence intervals, and is most commonly applied when the estimates are independent (hence uncorrelated). It can be shown that the approach of comparing overlap between confidence intervals is equivalent to making the conservative assumption that ρ = −1; see [6]. In contrast, in that setting, our proposed method differs and is less conservative since the upper bound for the standard error would be based on the assumption that ρ = 0.

Finally, we note that there are some measures, such as the population attributable risk [4], that cannot be formulated as differences in two parameters; in the Appendix we extend the results in Section 2 of the Methods for such non-linear functions of two parameters. The method can also be applied in the meta analysis of a general function of two parameters, say g(*β*_1_,*β*_2_), when for one or more of the studies only point estimates and standard errors are available for *β*_1_ and *β*_2_. The usual fixed effect meta-analytic estimator is simply a weighted average, with weights that are the inverse of the variance (or squared standard errors). The method described in the Appendix can be used to obtain an upper bound for the standard error, and hence the weight, when only point estimates and standard errors are available for *β*_1_ and *β*_2_.

## Appendix

### Confidence Intervals for General Non-Linear Functions

Suppose we are interested in a general non-linear univariate function of the two parameters, say g(*β*_1_,*β*_2_), which cannot be written as a difference *β*_1_−*β*_2_. Because of the conservativeness of the Bonferroni confidence interval in Section 1 of the Methods in comparison to the alternative confidence interval proposed in Section 2, here we discuss confidence intervals similar to those in Section 2 for a general non-linear function. That is, we consider 95% confidence intervals of the form 
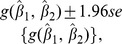
 using a conservative estimate for 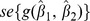.

Using the so-called “delta method”, 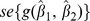 can be approximated as 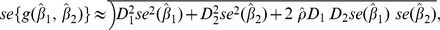
where *D_1_* is the derivative of 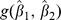 with respect to 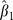 and *D_2_* is the derivative of 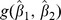 with respect to 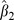, and 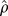 is again the (unreported) estimated correlation between 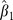 and 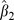.

For example, consider the population attributable risk (PAR), 
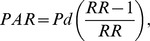
where *Pd* is the probability of exposure given disease, and *RR* is the multivariate relative risk. For the PAR, with *β*_1_ = *Pd* and *β*_2_ = *RR* the delta method gives, 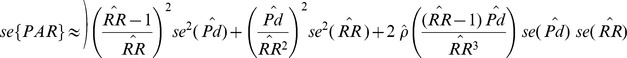,where 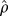 is the estimated correlation between 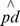 and 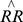. From published results, one can easily obtain all the estimates [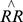,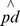, 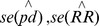] in 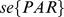, so that one would again choose the value of 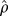 that gives the maximum value of 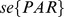. If 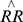
≥1, then one would choose 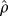  = 1; if 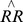 <1, then one would choose 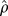 = −1. Finally, we note that the Bonferroni method can also be used to obtain confidence intervals for the PAR; see [4]. Both methods ensure coverage probability of at least 95%. However, as discussed in Section 2 of the Methods, the use of an upper bound for the standard error of the difference, *β*_1_−*β*_2_, yields narrower confidence intervals than the Bonferroni method; we conjecture that this result also holds for non-linear functions of the two parameters such as the PAR.
